# Teachers’ impact on dental students’ exam scores in teaching pathology of the oral cavity using WSI

**DOI:** 10.1186/1746-1596-8-S1-S25

**Published:** 2013-09-30

**Authors:** Janusz Szymas, Mikael Lundin, Johan Lundin

**Affiliations:** 1 Department of Clinical Pathology, Poznan University of Medical Sciences. Przybyszewski Str. 49. 60-355 Poznan, Poland; 2 Institute for Molecular Medicine Finland FIMM, P.O. Box 20, FN-00014, University of Helsinki, Finland

## Background

There is an overall increasing tendency at medical universities around the world to digitize microscope histopathological slides from teaching collections for web-based studies [1]. For the last seven academic years (2005 - 2012), we have developed and evaluated a user-friendly on-line interactive teaching and examination system to facilitate student access to Pathology of the Oral Cavity course material, including histological laboratory specimens. It made microscope laboratory studies in oral cavity pathology more efficient and teaching resources have become available for interactive use from anywhere at any time, independent of class schedules. The WebMicroscope allows students to independently explore entire histological slides. Students control the content and the rhythm of delivery of the content. Digital slides produced using whole slide imaging (WSI) can be visualized at any magnification and moved in the x-y axis, which perfectly imitates using a traditional microscope and a glass slide [2].

Examination which applies WSI technology on-line requires a sophisticated test management software. In the past, we used an examination system that was restricted to using standard static images. This was technically less challenging and followed a routine which was similar to that of conventional paper-based examinations [3,4]. WSI is more demanding as it requires the use of innovative image serving and viewing software and, because of the enormous size of the images, a storage facility capable of storing hundreds of gigabytes. However, it reflects much better the usual laboratory activities [5]. Using WSI allows asking questions about the overall diagnosis without specifically highlighting the key diagnostic features as it must be done in the field of view of a static image. Even if a question refers to a specific field of view, the student can zoom out and see that field in the overall context of the slide. This contextual information easily available in virtual microscopy is essential in teaching students both how to find the key diagnostic fields within a whole slide and to perceive how a diagnostic field fits into the surrounding non-diagnostic slide context. Our first seven-year experience in developing and using an examination system which applies WSI indicates that configuring an on-line practical exam in Pathology of the Oral Cavity requires flexibility over the question types that can be asked.

Our practical examination in oral pathology is scheduled at the end of the 3rd year dental curriculum of students in the Department of Clinical Pathology at the University of Medical Sciences in Poznan, Poland. Because the number of new students enrolling every academic year is high, students work in groups which are supervised by different teaching assistants with heterogeneous teaching experience. The aim of this study was identifying if in such a context the teacher has still an impact on the students’ exam scores.

## Material and methods

Each practical exam consisted of 50 multiple-choice questions per student. Every question was displayed together with the adequate whole slide image and students had to provide an answer based on the interpretation of this slide. The scope of whole slide images were broad, designed to include all slides used during the laboratory practicals in the Pathology of the Oral Cavity. Questions and whole slide images were presented in a different order to every student. For the examination in the academic years 2008 - 2012, we used 50% of randomly rotated whole slide images to prevent “fossilization” of the students. Students were shown how to use the software at the beginning of the practical exam and were given unlimited time to complete the exam. There was no time limit per slide either. Students could also view slides or answer questions in any order. All sessions of the practical exam using WSI took place in a secure location, the computer laboratory at the Department of Pathology, and were proctored all the time by teaching assistants. All examinations were graded using the same scale. Participants were all dental students enrolled to the Pathology of the Oral Cavity course between 2005 and 2012.Students were also required to complete a short anonymous survey at the time of the exam.

## Server architecture

The WebMicroscope was used in the current examination system to host, manage, and deliver WSI-based digital slides to the students during the practical examination. In this context, a dedicated Web viewer interacts with an image server, which serve out appropriate regions of the slide image. In this way, students can view whole slides in real time without the need to download entire images. For the practical examination in the Pathology of the Oral Cavity, multiple image servers operated at least in tandem (Poznan, Helsinki) allowing to process multiple requests for images per second.

## Client module software

The client module is responsible for presenting data to the student. It is the only part of the system visible to the user and consists mainly of an independent application which communicates with the other modules of the system through well-defined interfaces. The messages of the front-end virtual-slide viewer were written based on a native, downloadable browser plug-in, or java script based dynamic HTML. In the latter case, it does not require any specific downloads to the client work station.

## Statistical analysis

The results from all students were collected and analysed automatically by the Examination module and the Statistic module of the examination system software. Already at the end of the examination session, the administrator could download results for single participants, groups of students, or the whole cohort. In parallel the software automatically compiled the data on chosen answers to multiple-choice questions and performed statistical analysis on them. Here we provide descriptive statistics and t-test analysis using the analysis dataset from Pathology of the Oral Cavity examinations.

## Results

Students were surveyed regarding their preferences for the use of virtual microscopy in the curriculum based on their first exposure to this technology. The survey results indicate that an overwhelming majority of students found the WebMicroscope system relatively easy to use and helpful. Their answers to the question about the usefulness of the WSI-based examination system (rating from 0 to 10) were constant throughout the seven years of this study. The lowest mean value (8.4 ± 1.4) was recorded in 2006 and the highest (9.1 ± 1.7) in 2009 and later, after some improvements.

In the first examination, in 2006, a cohort of 8 students scored with a 98% ± 2.7%. Similar result was obtained one year later with a cohort of 84 students ( 97,2% ± 4.7%). At this time, 50 whole slide images were used in 2006 and 64 whole slide images in 2007 during laboratory practices and during the practical examination. With a growing number of whole slide images used in the subsequent years (81 whole slide images used in 2008 and 125 in 2011), the rate of correct students’ answers to the exam questions was only a little bit lower - circa 94% (Table 1). In the last four academic years (2008 – 2012), we used 50% of randomly rotated whole slide images. The rotation did not influence the performance of the students.

**Table 1:** Computer-administered practical exam performance using WSI

Year		2006	2007	2008	2009	2010	2011
**Number of students**		8	84	92	96	101	83

**Number of questions**		50	64	50	50	50	50

**Number of whole slide images**		50	64	81	82	103	125

**Correct students’ answers**	**AVG**	98%	97.2%	92.1%	93.8%	93.4%	93.9%
	
	**STDEV**	2.7%	4.7%	4.4%	5.6%	8%	5.2%
	
	**RANGE**	88% - 100%	73% - 100%	76% - 100%	78% - 100%	44% - 100%	74% - 100%

When analyzing the students’ scores by student groups supervised by different teaching assistants, we found that the mean exam results were different between the groups. There were some student groups which scored high and some groups with lower grades (Table 2). Differences between these student groups were statistically significant (P-value < 0.050) in all academic years except for 2009-10. Unfortunately, the rotation of teaching assistants in our Department is high but during the period of this study we could identify at least three teachers whose groups have permanently scored high or the highest during the exam. We have also noted a couple of unsuccessful teaching assistants.

**Table 2:** Students’ scores by students groups.

Year	Students’ group	Number of students	Correct students’ answers				Number of students with all corrected answers
				
			AVG	STDEV	MIN	MAX	
2009	I	15	44.60	4.64	33	49	-

	II	16	47.18	1.97	43	50	1

	III	16	47.50	2.19	44	50	4

	IV	17	48.41	2.12	44	50	6

	V	16	46.19	2.53	41	50	2

	VI	16	47.31	1.49	45	50	3

2010	I	18	47.44	2.93	39	50	2

	II	14	47.67	2.29	42	50	4

	III	15	45.27	4.54	37	50	2

	IV	17	47.23	3.17	38	50	3

	V	20	45.55	6.37	22	50	3

	VI	17	46.70	2.52	40	50	1

2011	I	14	45.14	3.57	37	49	-

	II	13	48.38	1.26	45	50	2

	III	12	47.58	1.44	46	50	2

	IV	15	48.20	2.18	42	50	4

	V	15	46.13	3.00	38	50	1

	VI	14	46.14	2.98	39	50	2

## Discussion

Testing large numbers of students in the exam context with many virtual slides is a challenging operation with many potential bottle necks. To overcome some of them, whole slide images were stored on an independent server. Serving these slides as part of the practical examination is bandwidth and server intense, particularly if the image latency is to be kept to a minimum with many students accessing the images simultaneously. A successful examination system requires multiple image servers which are carefully load balanced which implies that, when one server gets busy due to user activity, another server takes over and provides the services that are being requested. For this practical examination in the Pathology of the Oral Cavity, multiple image servers operated at least in tandem (Poznan, Helsinki) allowing processing multiple requests for images per second. The fine focus feature, the small overview slide [6] and the possibility to use the WebMicroscope to view the whole slide images were all perceived favorable, and most students did not notice any delays during examination. More than 90% of responders found the WSI-based laboratory practicals and the practical exam useful and helpful to improve their understanding of Pathology of the Oral Cavity. At practical examinations, the students gave from 94% to 98% correct answers. So there is clear evidence of learning benefits from using WSI. Despite widely available self-study possibilities for the students, we were able to demonstrate differences between student groups. High and low scoring in student groups was associated with particular teaching assistants.

## Conclusions

Despite all technical problems, the WebMicroscope system is a promising tool for practical student examination using WSI during laboratory practicals in the Pathology of the Oral Cavity. With all the people involved working hard to solve these technical problems and preparing every year exam, we are confident that testing using WSI is going to play an essential role in the future of the university education and examinations. Nevertheless, despite widely available self-study possibilities, good teachers still create a substantial value. Exam scores are helpful in identifying such teachers. This study also shows evidence that existing measures are informative about the teachers’ impact on student scores.

## Abbreviations used

WSI: whole slide imaging

## Competing interests

The authors have no competing interests.

## Authors contributions

JS organised and managed the study and drafted the manuscript. ML was in charge of data management, managed WebMicroscope server and software and assisted in the manuscript, JL provided advice and expertise, and assisted in the manuscript.
